# Effectiveness of Mobile Apps in Promoting Healthy Behavior Changes and Preventing Obesity in Children: Systematic Review

**DOI:** 10.2196/34967

**Published:** 2022-03-28

**Authors:** Kiana W Yau, Tricia S Tang, Matthias Görges, Susan Pinkney, Annie D Kim, Angela Kalia, Shazhan Amed

**Affiliations:** 1 Department of Medicine The University of British Columbia Vancouver, BC Canada; 2 Research Institute BC Children's Hospital Vancouver, BC Canada; 3 Department of Anesthesiology Pharmacology and Therapeutics The University of British Columbia Vancouver, BC Canada; 4 Department of Pediatrics The University of British Columbia Vancouver, BC Canada

**Keywords:** childhood obesity, mobile health, health behaviors, prevention, study design, systematic review, mobile phone

## Abstract

**Background:**

Mobile apps have been increasingly incorporated into healthy behavior promotion interventions targeting childhood obesity. However, their effectiveness remains unclear.

**Objective:**

This paper aims to conduct a systematic review examining the effectiveness of mobile apps aimed at preventing childhood obesity by promoting health behavior changes in diet, physical activity, or sedentary behavior in children aged 8 to 12 years.

**Methods:**

MEDLINE, Embase, PsycINFO, CINAHL, and ERIC were systematically searched for peer-reviewed primary studies from January 2008 to July 2021, which included children aged 8 to 12 years; involved mobile app use; and targeted at least one obesity-related factor, including diet, physical activity, or sedentary behavior. Data extraction and risk of bias assessments were conducted by 2 authors.

**Results:**

Of the 13 studies identified, most used a quasi-experimental design (n=8, 62%). Significant improvements in physical activity (4/8, 50% studies), dietary outcomes (5/6, 83% studies), and BMI (2/6, 33% studies) were reported. All 6 multicomponent interventions and 57% (4/7) of standalone interventions reported significant outcomes in ≥1 behavioral change outcome measured (anthropometric, physical activity, dietary, and screen time outcomes). Gamification, behavioral monitoring, and goal setting were common features of the mobile apps used in these studies.

**Conclusions:**

Apps for health behavior promotion interventions have the potential to increase the adoption of healthy behaviors among children; however, their effectiveness in improving anthropometric measures remains unclear. Further investigation of studies that use more rigorous study designs, as well as mobile apps as a standalone intervention, is needed.

## Introduction

An estimated 150 million children worldwide currently live with obesity, and this number is projected to increase to 254 million by 2030 [1]. Childhood obesity, which tends to persist into adulthood [2], is one of the most pressing public health challenges of the 21st century. It is associated with an increased risk of developing lifelong chronic conditions such as type 2 diabetes, hypertension, and cardiovascular disease [3], as well as psychosocial consequences such as depression and anxiety [4].

Concurrent with the rising rates of childhood obesity, the adoption of mobile devices, such as smartphones and tablets, and the use of mobile apps on these devices, have also increased among children of all ages [5]. For example, in the United States, rates of smartphone ownership among children aged 8 to 12 years and 13 to 18 years have grown substantially from 25% to 41% and from 67% to 84%, respectively, from 2015 to 2019 [5]. Furthermore, 41% and 52% of Canadian children, aged 9 to 11 years and 12 to 14 years, respectively, reported playing games or using apps on electronic devices at least 5 days a week [6]. Owing to the increasing popularity of mobile devices and apps, many health and fitness apps targeting key modifiable risk factors such as diet, physical activity, and reduction of sedentary behavior have been developed and used in health promotion interventions for children [7]. These interventions tend to be (1) based on at least one behavioral change theory; (2) targeted at ≥1 behavioral, anthropometric, psychological, or process outcomes; and (3) multicomponent, where mobile apps are used in addition to other intervention components such as physical games, food or physical activity diaries, wearable technology, and SMS text messaging [8].

To date, studies have shown mobile apps have a promising role in increasing motivation and promoting goal-setting behavior to address childhood obesity [9]. Multicomponent intervention bundles involving mobile apps appear to be more effective than standalone mobile app interventions in addressing behavioral outcomes such as diet, physical activity, and sedentary behavior [10]. However, the results from these studies have generally been inconsistent, and the efficacy (performance of an intervention under ideal circumstances) and effectiveness (performance of an intervention in real-life conditions) of mobile apps in delivering interventions to address childhood obesity remain unclear [11]. Most studies have focused on investigating the feasibility, usability, and acceptability of mobile health interventions rather than assessing efficacy and effectiveness via controlled trials [8].

Systematic reviews have focused on mobile health interventions that target diet, physical activity, and sedentary behavior, which are factors associated with childhood obesity; however, most examined mobile apps in combination with other interventions such as exergames (digital games that involve physical movements for active gameplay), video games, websites, and SMS text messaging [12-15]. Of the few reviews that focused solely on mobile apps, most involved adolescents [9,15] or a mix of pediatric and adult populations [10]. Therefore, there is a knowledge gap in the literature on the effectiveness of mobile health technologies that promote healthy behavior change to prevent childhood obesity in school-aged children (8 to 12 years), which is a critical period for children to develop positive habits and behaviors as they form their own identities. The objective of this study is to conduct a systematic review to examine the effectiveness of mobile apps that promote healthy behavior changes in diet, physical activity, or sedentary behavior in children aged 8 to 12 years.

## Methods

### Literature Search

This systematic literature review was conducted and is reported according to the PRISMA (Preferred Reporting Items for Systematic Reviews and Meta-Analyses) statement [16]; the protocol was not preregistered in any database. Medical Subject Heading terms and keywords related to (1) mobile app development, (2) obesity prevention and healthy behaviors, and (2) mixed methods research interventions were identified with guidance from a research librarian (CP). The search strategy was designed such that the results contained at least one search term from each of these 3 categories. Using this strategy, the electronic databases MEDLINE, Embase, PsycINFO, CINAHL, and ERIC were searched in July 2021 to identify records published between January 2008 and June 2021. The year 2008 was selected as the lower limit of publication years as it coincides with the launch of both the Android market [17] and Apple App Store [18], which are platforms for users to download apps on their digital devices. To retrieve pediatric articles, search filters were used for MEDLINE [19], Embase [20], and CINAHL [21], whereas age groups and education level limiters were used for PsycINFO and ERIC, respectively. The complete search strategy is presented in Multimedia Appendix 1. Gray literature was searched by screening the reference lists of the included articles, research studies listed in the US National Library of Medicine clinical trials database (using search terms *Obesity*, *Childhood*, and *Mobile* *health*), the first 100 results from a search of keywords *childhood obesity* and *mobile* *health* on Google Scholar, and results from the title and abstract search of ProQuest Dissertations and Theses Global with search terms *childhood obesity* and *mobile*. Only peer-reviewed studies resulting from the gray literature search were considered.

### Eligibility Criteria

The eligibility criteria for articles included (1) peer-reviewed primary studies written in English; (2) published between January 2008 and the end of June 2021; (3) children aged 8 to 12 years as participants (studies with children participants outside the age range but with some within the target age range were deemed eligible); (4) the use of a mobile app by children and their immediate caregivers; and (5) targeting behavior change in at least one obesity-related factor, including diet, physical activity, or sedentary behavior. Participants of all health statuses—healthy weight, at risk, or with obesity or overweight—were considered. To provide a broad overview of the current published literature, experimental (eg, randomized controlled trial [RCT]), quasi-experimental, observational, and mixed methods studies were included. Articles that described only the use of websites, email, or SMS text message–based interventions were excluded.

### Study Selection

After removing duplicates, a single author (KWY) performed an initial screening based on the title and abstract to identify full-text articles for assessment of eligibility. Any uncertainty that arose from this process was discussed with SA, and decisions were made by consensus. Articles that could not be excluded based on the information provided in the title and abstract were included in the full-text review. KWY and AK then reviewed the full-text articles independently, after which they compared their decisions on eligibility, discussed and resolved any discrepancies by consensus, and finalized the list of articles to be included in this review.

### Data Extraction and Quality Assessment

Information on study design, inclusion criteria, sample size, sociodemographic characteristics of participants, study details (eg, behavior change theory and study length), description of the mobile app, and outcome measures (eg, anthropometry, physical activity, diet, screen time, sedentary behavior, and process evaluation) were independently extracted by KWY, AK, and ADK, following a predetermined data extraction template developed by KWY based on interventions and outcomes identified during the development of the research aim, eligibility criteria, and search strategy (Multimedia Appendix 2). Discussions between KWY, AK, ADK, and SA (as needed) occurred regularly to reach a consensus in cases of disagreement. KWY and ADK independently assessed the risk of bias of the included studies using the Cochrane Risk of Bias 2 tool for randomized trials [22] and the Risk of Bias in Nonrandomized Studies of Interventions tool for observational and quasi-experimental studies [23]. Any discrepancies in the ratings were resolved via discussion between the authors until a consensus was reached.

## Results

### Study Characteristics

**Figure 1:**
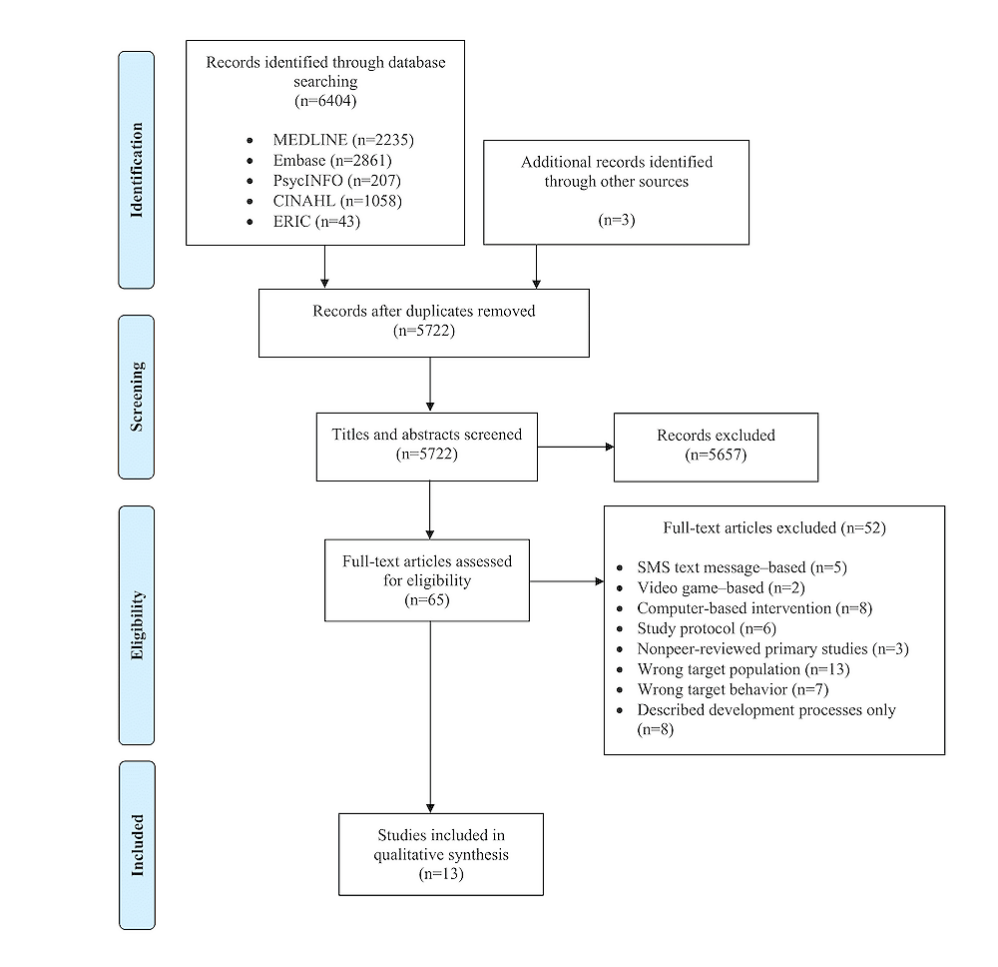
PRISMA (Preferred Reporting Item for Systematic Reviews and Meta-Analyses) flow diagram summarizing the study selection process.

A total of 13 studies met the eligibility criteria (Figure 1), of which 8 (62%) were from the United States [24-31], and the remaining 5 (38%) studies were from Australia [32], Canada [33], the Netherlands [34], New Caledonia (Overseas France) [35], and Portugal [36]. The number of participants per study ranged from 18 to 2477, with 15% (2/13) of studies including only male [32] or only female [27] participants. The age of the participants ranged from 4 to 21 years, with 46% (6/13) of studies involving only adolescents (aged >10 years) [25,28,32,34-36]. Across the studies, there were diverse representations from various racial or ethnic minority groups, including African [31,32], African American [25-27,30], American Indian or Alaska native [27,31], Asian [26,32], Hispanic [25,26,31,32], Pacific Islander [27,35], and Middle Eastern [32]. In 38% (5/13) of studies, more than half of the study participants were from racial or ethnic minority populations [25-27,29,31]. Targeted recruitment of participants from low socioeconomic backgrounds was conducted in 38% (5/13) of studies [27,29-32]. Approximately 15% (2/13) of studies included only participants who were at risk for developing obesity, as determined by their failure to meet international physical activity or screen time guidelines [32] and positive results on a food addiction scale [25]. Of the 13 studies, 4 (31%) were randomized intervention studies [27,28,32,34].

### Risk of Bias Assessment

Of the 4 randomized intervention studies included, 3 (75%) were rated as having some concerns regarding their overall bias [27,28,34], whereas 1 (25%) study had a low risk of bias [32] (Table 1). A potential major source of bias in the 31% (4/13) of studies that used an RCT design [27,28,32,34] was from the randomization process itself; 75% (3/4) of studies [23,24,28] did not elaborate on the randomization methods other than providing a statement that the study was randomized. Owing to the nature of the interventions, blinding of participants and those delivering the intervention from group allocation were impossible for all studies. Of the 4 RCT studies, 1 (25%) attempted to blind assessors from treatment allocations but were only successful at baseline and not at follow-up [32]. Only 75% (3/4) of RCT studies reported incomplete outcome data because of participant absence on the day of data collection [32,34], loss to follow-up [27,32], withdrawal from the study [32], and malfunctioning of the measuring devices [34]. 50% (2/4) of RCT studies reported objective measures, such as BMI and step count, as primary outcomes [32,34]. The prespecified intentions for data analysis were only available for 50% (2/4) of the RCT studies in the form of a clinical trial register [37] and a published protocol [38].

Of the 9 nonrandomized intervention studies, 5 (56%) were assessed as having a moderate risk of bias [24,30,31,33,35] and 4 (44%) as having a serious risk of bias [25,26,29,36] (Table 2). Baseline confounding was found to be a serious risk of bias in 33% (3/9) of studies, with 67% (2/3) of studies measuring but not controlling for potential confounding factors [25,29] and 33% (1/3) of studies neglecting to consider previous exposure to interventions as a potential confounder for a small subset of participants in a retrial [26]. Of the 9 studies, all but 1 (11%) study [36] scored a low or moderate risk for missing data. A prespecified analysis plan was available for 22% (2/9) of studies in the form of a trial register [39] and study protocol [40].

**Table 1:** Risk of bias assessment scores for randomized trials.

Study	Randomization process	Deviations from intended interventions	Missing outcome data	Measurement of the outcome	Selection of the reported result	Overall bias
Byrne et al [28]	Some concerns	Low	Low	Low	Some concerns	Some concerns
Nollen et al [27]	Some concerns	Low	Some concerns	Low	Some concerns	Some concerns
Smith et al [32]	Low	Low	Low	Low	Low	Low
van Woudenberg et al [34]	Some concerns	Low	Low	Low	Low	Some concerns

**Table 2:** Risk of bias assessment scores for nonrandomized trials.

Study	Confounding	Selection of participants	Intervention classification	Deviations from intended intervention	Missing data	Measurement of outcomes	Selection of reported result	Overall bias
Bell et al [31]	Moderate	Low	Low	Low	Low	Moderate	Moderate	Moderate
Blackman et al [29]	Serious	Low	Low	Low	Moderate	Low	Moderate	Serious
Dunton et al [26]	Serious	Low	Low	Low	Low	Low	Moderate	Serious
Galy et al [35]	Moderate	Moderate	Low	Low	Moderate	Low	Moderate	Moderate
Patten et al [33]	Moderate	Low	Low	Low	Low	Low	Low	Moderate
Pretlow et al [24]	Moderate	Low	Low	Low	Low	Moderate	Moderate	Moderate
Sousa et al [36]	Moderate	Low	Low	Low	Serious	Moderate	Low	Serious
Struempler et al [30]	Moderate	Low	Low	Low	Moderate	Moderate	Moderate	Moderate
Vidmar et al [25]	Serious	Low	Moderate	Low	Low	Low	Low	Serious

### Study and Intervention Design

Table 3 outlines the study and the intervention design features. Quasi-experimental study designs were the most prevalent (8/13, 62%) and included within-subject design (1/8, 13%) [33], one-group posttest-only design (1/8, 13%) [26], and pretest–posttest designs (6/8, 75%) [24,25,29-31,36]. The remaining 38% (5/13) of studies were cluster RCTs [32,34], RCTs [27,28], and an exploratory study [35] (which was used during a preliminary investigation of a research question with minimal available published evidence). The intervention duration ranged from <1 month (5/13, 38%) [26,28,31,33,34], with one of the studies testing a suite of imagination-based mobile games lasting 1 hour [33]; between 1 month and 3 months (3/13, 23%) [27,29,35]; and between 3 and 6 months (5/13, 38%) [24,25,30,32,36]. Only one of the studies included a follow-up assessment to determine the sustainability of changes 8 months after the end of a 20-week intervention [32]. Of the 13 studies, 2 (15%) were treatment interventions for overweightness or obesity [24,25], 3 (23%) were obesity prevention interventions [27,30,32], and 8 (62%) were healthy behavior promotion interventions [26,28,29,31,33-36]. The targeted healthy behaviors included physical activity [26,29,31-35], screen time or sedentary behavior [26,29,32], and nutritional intake [24,25,27,28,30-32]. Most studies targeted only a single healthy behavior. All studies included apps developed solely for the purpose of their intervention. Of the 13 studies, 11 (85%) included the use of only a single app, whereas 1 (8%) study used a collection of 4 apps [29], and another used a collection of 7 apps [30] as part of the intervention.

**Table 3:** Study design and intervention type of studies included.

Study	Study design									Intervention type							Multicomponent			Duration			Behavior change theory
	Quasi-experimental				RCT^a^		Exploratory		Healthy behavior promotion			Obesity prevention		Obesity treatment									
	Within-subject	One-group posttest-only	Pretest-posttest																				
Bell et al [31]			✓ (control)					✓							✓			3 weeks			Self-determination theory; social cognitive theory		
Blackman et al [29]			✓					✓										6 weeks			Fogg Behavior Model		
Byrne et al [28]				✓				✓										9 days			Social cognitive theory		
Dunton et al [26]		✓						✓										1 day			N/A^b^		
Galy et al [35]						✓		✓										4 weeks			N/A		
Nollen et al [27]				✓							✓							12 weeks			Behavioral weight control principles		
Patten et al [33]	✓							✓										1 hour			N/A		
Pretlow et al [24]			✓										✓		✓			20 weeks			Addiction treatment model		
Sousa et al [36]			✓ (control)					✓							✓			6 months			N/A		
Smith et al [32]				✓ (cluster)							✓				✓			20 weeks			Social cognitive theory; self-determination theory		
Struempler et al [30]			✓								✓				✓			17 weeks			Experiential learning theory		
Vidmar et al [25]			✓										✓		✓			26.1 weeks			Addiction treatment model		
van Woudenberg et al [34]				✓ (cluster)				✓										1 week			Self-determination theory		

^a^RCT: randomized controlled trial.

^b^N/A: not applicable.

### Behavior Change Theory

Of the 13 studies, all but 4 (31%) studies [26,33,35,36] used a behavior change theory as the foundation for app and intervention design to promote healthy behavior change among participants (Table 3); 8 different behavior change theories were reported, with 7 (54%) studies using 1 behavior change theory [24,25,27-30,34], and 2 (15%) studies combining 2 behavior change theories [31,32]. Approximately 23% (3/13) of interventions [28,31,32] used social cognitive theory [41], which suggests that learning and acquiring certain behaviors occur through reciprocal interactions between individuals and their environment. Another 15% (2/13) of studies [24,25] used the same app and adopted an addiction treatment model [42]. Other behavior change theories included the self-determination theory [31,32,34], behavioral weight control principles [27], the Fogg Behavior Model [29], and the experiential learning theory [30].

### App Design Features

The most common app design feature was gamification (7/13, 54%), whereby healthy behavior promotion was integrated into the app via digital pets [28], simulated gardening [31], on-screen instructions for individual and small-group play [29,33], and team challenges [35]. User progress was rewarded through song-based rewards [27], a wall of fame [36], and augmented reality trophies [31]. Another common design feature was behavioral monitoring (6/13, 46%), which included self-reporting via questionnaire administration [24-26,34]; self-monitoring of healthy behaviors and anthropometric measures [36]; and collection of information from accelerometers [32,34], food scales [24,25], and body weight scales [24,25]. Another common feature was goal setting (5/13, 38%) for physical activity [32,35]; screen time [27,32]; fruits and vegetables [27]; sugar-sweetened beverages [27]; and the reduction in food amounts, food problems, and snacking [24,25]. Push notifications [32,34], SMS text messages [24,25], and emails [28] were used to deliver tailored motivational messages. When a participant experienced excessive weight loss, one of the apps automatically alerted the research team [24,25]. Approximately 38% (5/13) of studies representing 4 apps incorporated social support features, including peer assessments [32], peer nominations for influence agents [34], app bulletin boards [24,25], discussion forums [36], and in-app chat groups [24,25,36]. A total of 2 apps from 23% (3/13) of studies [24,25,28] allowed users to take photographs of their meals in the app and submit them to a research server for review and scoring.

### Outcome Measures

A summary of the reported outcome measures is presented in Table 4 and Table 5. Approximately 62% (8/13) of studies reported significant improvements in at least one of the measured healthy behaviors. Measures of physical activity were the most commonly reported outcomes in the intervention studies [26,29,32-36]. Of the 7 studies that included physical activity as an outcome, 4 (57%) reported statistically significant increases in physical activity levels following app use, as measured by moderate to vigorous physical activity (MVPA) via estimation from heart rate [33] and accelerometry [26,32], number of steps [26,32], and metabolic equivalents as determined by accelerometry [29]. Participants who used Biba Games, a suite of mobile apps aimed at encouraging imagination-based outdoor play via playful directives, displayed greater amounts of MVPA than regular playground gameplay, as demonstrated by a significant increase in heart rate measured in beats per minute (mean change 17.8, SD 28.3%; *P*<.05) [33]. In a 6-week smartphone game–based app program aimed at promoting physical activity in an afterschool program, participants achieved greater metabolic equivalents during gameplay with mobile app games than with nonguided free play (*P*=.02) [29]. The investigation of the acceptability and validity of a 4-day ecological momentary assessment protocol using mobile surveys to measure physical activity and sedentary behavior in children revealed significantly higher step counts (*P*<.001) and the likelihood of ≥5 minutes of MVPA (*P*<.001) during ecological momentary assessment–reported physical activity [26]. Finally, in Active Teen Leaders Avoiding Screen-time (ATLAS), which was a 20-week multicomponent obesity prevention intervention using smartphone technology, significant intervention effects were found for muscular fitness (mean 0.90, SE 0.49 repetitions; *P*=.04) and resistance training skills (mean 5.70, SE 0.67 units; *P*=.001) [32].

Dietary outcomes were reported in 46% (6/13) of intervention studies and included fruit and vegetable intake [27,30,31], sugar-sweetened beverage intake [27,31,32], the likelihood of eating breakfast [28], self-efficacy toward fruit and vegetable consumption [31], and attitude toward healthy eating [36]. Of the 4 studies that measured at least one dietary intake outcome, 2 (50%) reported significant improvements in fruit and vegetable intake [30] and sugar-sweetened beverage consumption [32], both of which were measured using self-reported questionnaires. Body Quest: Food of the Warrior [30] is a multicomponent elementary school–based childhood obesity prevention program aimed at increasing fruit and vegetable consumption, increasing physical activity, and promoting family involvement via a mix of traditional curriculum teaching, iPad app–based education, weekly fruit and vegetable tastings, and weekly take-home activities. Intervention participants demonstrated significant increases in fruit (*P<*.01) and vegetable (*P*<.001) consumption over the course of the program, increasing from 7 to 8 weekly fruit and vegetable servings in total. At the end of the program, participants consumed significantly more weekly servings of fruits (*P*<.001) and vegetables (*P*<.001) than the control group. In ATLAS [32], participants demonstrated a significant reduction in sugar-sweetened beverage consumption, as measured by the number of glasses per day (mean 0.60, SE 0.26 glasses per day; *P*=.01), after the 20-week intervention. The use of a mobile app as a standalone childhood obesity prevention tool resulted in a mix of nonsignificant and significant intervention effects. In a 12-week mobile technology intervention for obesity prevention among girls of diverse racial and ethnic backgrounds [27], participants tested a mobile app that facilitated goal setting, self-monitoring, and positive reinforcement to promote healthy behaviors. A 24-hour dietary recall failed to detect any significant improvements in fruit and vegetable consumption and sugar-sweetened beverage consumption. However, participants who tested a digital pet mobile game app aimed at improving eating behaviors demonstrated a significant increase in their likelihood of consuming breakfast (*P*<.05) [28]. All 23% (3/13) of studies that measured changes in the perception of healthy diet practices reported significant improvements, including adopting a more positive perception toward healthy dietary changes [28,31,36] and an increased likelihood of consuming breakfast [28].

**Table 4:** Measured anthropometry and physical activity outcomes and effect size of included studies.

Study		Anthropometry						Physical activity				
		BMI	BMI *z* score	BMI percentile	Waist circumference	Body fat percentage	MVPA^a^		Step count	MET^b^	Physical strength or fitness	Attitude or perception
**Bell et al [31]**												
	Significance		NS^c^	NS								
	Statistics		*P*=.30	*P*=.32								
**Blackman et al [29]**												
	Significance									✓		
	Statistics									*P*=.02		
**Byrne et al [28]**												
	Significance											
	Statistics											
**Dunton et al [26]**												
	Significance						✓		✓			
	Statistics						*P*<.001		*P*<.001			
**Galy et al [35]**												
	Significance						NS		NS		NS	NS
	Statistics											
**Nollen et al [27]**												
	Significance	NS										
	Statistics	*d*=0.03; *P*=.91										
**Patten et al [33]**												
	Significance						✓					
	Statistics						*d*=0.53; *P*<.05					
**Pretlow et al [24]**												
	Significance			✓								
	Statistics			*P*<.01								
**Sousa et al [36]**												
	Significance											NS
	Statistics											η_p_^2^=0.01; *P*=.19
**Smith et al [32]**												
	Significance	NS			NS	NS	NS		NS		✓	
	Statistics	*P*=.84			*P*=.16	*P*=.99	*P*=.14 (weekday); *P*=.80 (weekend)		*P*=.41 (weekday); *P*=.57 (weekend)		*P*=.04 (muscular fit); *P*<.001 (RT^d^)	
**Struempler et al [30]**												
	Significance											
	Statistics											
**Vidmar et al [25]**												
	Significance		✓	✓								
	Statistics		*P*<.001	*P*<.001								
**van Woudenberg et al [34]**												
	Significance								NS			
	Statistics								*P*=.66			

^a^MVPA: moderate to vigorous physical activity.

^b^MET: metabolic equivalent.

^c^NS: nonsignificance.

^d^RT: resistance training.

**Table 5:** Measured dietary, screen time, feasibility and process evaluation outcomes and effect size of included studies.

Study			Dietary									Screen time			Feasibility or process evaluation
			Fruits and vegetables		Sugar-sweetened beverages		Breakfast likelihood		Attitude or perception						
**Bell et al [31]**															
	Significance	NS^a^		NS				✓							
	Statistics	*P*=.41 (fruit); *P*=.38 (vegetable)		*P*=.75				*P*=.01							
**Blackman et al [29]**															
	Significance												✓		
	Statistics														
**Byrne et al [28]**															
	Significance					✓		✓					✓		
	Statistics					η_p_^2^=0.20; *P*<.05		η_p_^2^=0.23; *P*<.05							
**Dunton et al [26]**															
	Significance														
	Statistics														
**Galy et al [35]**															
	Significance												✓		
	Statistics														
**Nollen et al [27]**															
	Significance	NS		NS						NS					
	Statistics	*d*=0.44; *P*=.13		*d*=–0.34; *P*=.09						*d*=0.09; *P*=.76					
**Patten et al [33]**															
	Significance														
	Statistics														
**Pretlow et al [24]**															
	Significance												✓		
	Statistics														
**Sousa et al [36]**															
	Significance							✓							
	Statistics							η_p_^2^=0.03; *P*=.03							
**Smith et al [32]**															
	Significance			✓						✓			✓		
	Statistics			*P*=.01						*P*=.03					
**Struempler et al [30]**															
	Significance	✓													
	Statistics	*P*<.001 (fruit); *P*<.001 (vegetable)													
**Vidmar et al [25]**															
	Significance												✓		
	Statistics														
**van Woudenberg et al [34]**															
	Significance														
	Statistics														

^a^NS: nonsignificance.

BMI or BMI-derived measures were reported in 38% (5/13) of studies and included BMI [27,32], BMI *z* score (zBMI) [25,31], BMI percentile [31], and percentage over BMI relative to the 95th percentile (%BMI_p95_) [25] and 50th percentile (%BMI_p50_) [24], all of which were derived from height and weight data measured by trained research personnel. Of the 5 studies, 2 (40%) reported significant improvements [24,25]; both reported on the same weight loss intervention but in different settings—clinical [25] and community [24]—and measured various BMI-derived measures from baseline to program completion. The intervention was a multicomponent program based on an addiction treatment model that involved app use, as well as weekly phone meetings and group meetings to guide participants into staged, incremental food withdrawal to address problem foods, snacking, and meal size reduction. In the community setting, participants demonstrated a significant decrease in %BMI_p50_ from baseline to the end of the intervention (baseline: mean −0.051, SD 0.013 %BMI_p50_ per day; *P*<.01). In addition, participants in the health care setting [25] also experienced a significant decrease in %BMI_p95_ (coefficient=−0.02; 95% CI −0.03 to −0.01; *P*<.001), which is a more stringent measure, upon program completion compared with age-matched controls, with a significant decrease noted at 1, 3, and 6 months. Approximately 15% (2/13) of other studies that reported BMI only [27,32] found no significant differences in intervention completion despite comparable intervention durations (between 12 and 20 weeks).

Other reported measures included waist circumference [32], body fat percentage [32], strength and fitness measurements [32,35], recreational screen time [27,32], importance of eating healthy [28], attitudes and perceptions toward physical activity [35,36], and nutrition [31,36]. In an app-based digital pet intervention, when asked about the importance of healthy eating, participants who received only positive feedback from their digital pets (ie, happy pet avatar) reported viewing healthy eating as less important than those who received both positive and negative feedback (ie, sad pet avatar; *P*<.01), illustrating the motivational value of negative feedback [28]. Significant intervention effects were found for screen time (mean −30.00, SE 10.08 minutes per day; *P*=.03) in ATLAS [32]. Intervention participants of Virtual Sprouts, a 3-week intervention that involved a mobile gardening game and a classroom component, compared with the control group, achieved significant improvements in self-efficacy to eat (+1.6% vs −10.3%; *P*=.01) and cooking (+2.9% vs −5.0%; *P*=.05) fruits and vegetables [31]. A significant effect on the self-reported perception of nutrition (mean change 0.02, SD 0.48; *P*=.03) was reported among participants of TeenPower, a mobile health intervention aimed at promoting healthy behaviors in adolescence [36], compared with the control group (mean change −0.07, SD 0.42). No relationship was found between intervention effectiveness, as indicated by significant changes in reported outcome measures, and study quality, as assessed by the risk of bias assessment (Table 1 and Table 2).

### Feasibility and Process Evaluation

Feasibility and process evaluation data were reported in 46% (6/13) of studies. Of the 3 studies that examined program satisfaction via surveys [32] and semistructured focus groups [29], 3 (67%) reported high levels of satisfaction. Approximately 31% (4/13) of studies measured user enjoyment, of which 75% (3/4) reported high levels of participant enjoyment [28,29,35], and 25% (1/4) reported that only 44% of participants agreed that the intervention was enjoyable [32]. Other feasibility measures included ease of use, perspectives on app features [29], and sustained interest in the intervention [28,35]. Approximately 23% (3/13) of studies reported on process indicators, including compliance measured via attendance tracking [32], recruitment and retention rates [25], and facilitator rating of participant compliance [24,25].

## Discussion

### Principal Findings

Interventions that used mobile health apps and included children aged 8 to 12 years were effective in improving healthy behaviors associated with childhood obesity, such as diet, physical activity, and sedentary behavior, with 62% (8/13) of studies reporting significant positive changes in at least one healthy behavior outcome. However, there was a lack of strong evidence to suggest the effectiveness of these interventions in improving anthropometric measures, with only 40% (2/5) of studies, both describing the same intervention but performed in different settings (clinical and community), reporting at least one significant change in BMI *z* score [25] and BMI percentile [24,25]. This discrepancy between healthy behavior improvements and insignificant improvements in anthropometric measures may be accounted for by the use of different assessment methods. Except for physical activity, measures of healthy behaviors tended to be assessed by self-report questionnaires, which may be more prone to bias and inaccuracy than anthropometric outcomes, which are typically measured by trained research personnel.

Of the 13 studies included in this review, 8 (62%) described healthy behavior promotion interventions [26,28,29,31,33-36], which is indicative of the gradual shift in focus from treatment to preventive health. Although mobile apps have the potential to improve healthy behaviors, our review indicates that not all apps are equal in their effectiveness. Of the 12 apps included in this review, 9 (75%) apps (of the 13 studies, 10 (77%) studies represented these apps) reported significant results in ≥1 outcome measure [24-26,28-33,36]. Approximately 46% (6/13) of studies [24-26,28,29,33] found significant results in all outcome measures assessed and targeted no more than 2 outcomes, suggesting the increased effectiveness of apps with a narrow behavior change target. Automatic data collection [24,25,32] and gamification [27-29,31,33,35,36] were the key features of apps that resulted in effective interventions. Multicomponent interventions appear to be superior compared with standalone app interventions.

### Quasi-Experimental Designs Provide Flexibility for Proof-of-Concept Studies

Quasi-experimental study designs were the most common among the interventions described, with the one-group pre–posttest design being the most popular [24,25,29,30]. With a multitude of possible study designs (eg, interrupted time series and designs with or without control groups), quasi-experimental designs provide versatility, particularly in the context of limited resources. RCTs may require a large sample size and, as a result, greater amounts of resources such as funds and research personnel [43]. Researchers may have considered the unethical nature of performing randomization in at-risk populations, which could have been addressed by a stepped-wedge or waitlist study design but at the cost of a delay in treatment in the waitlist control group [44]. Quasi-experimental experiments can provide insight into correlation because of their design flexibility in the inclusion of retrospective control groups and multiple measures over time and can inform researchers whether it is worthwhile to conduct an RCT afterward to confirm causation [45].

### Behavioral Versus Adiposity Measures for Evaluation of Childhood Obesity Interventions

Although only 33% (2/6) of studies reported significant improvements in adiposity measures [24,25], 73% (8/11) of studies reported significant improvements in at least one healthy behavior outcome, such as physical activity and fruit and vegetable intake. This absence of significant improvements in adiposity measures in the presence of improvements in behavioral measures has been previously reported [12] and may be explained by the limited duration of the reported interventions, as many were considered proof-of-concept studies with limited resources. For instance, participants in the pilot study by Patten et al [33] engaged in only 2 separate 20-minute play sessions (with and without the use of the mobile app) separated by 10- to 15-minute breaks. Given the pilot nature of the study, the authors discussed the limited budget as a potential challenge and further acknowledged that the results may be insufficient to support the presence of meaningful interactions and generalizability to the population of interest. Using solely adiposity measures, such as BMI and other BMI-derived measures (eg, zBMI, %BMI_p95_, and %BMI_p50_), has been found to be insufficient for evaluating the effects of interventions for childhood obesity [46]. For example, results from a 9-week multicomponent, community-based childhood obesity intervention indicated that changes in zBMI were independent of changes in important health outcomes, such as cardiovascular fitness and physical activity, upon intervention completion [46].

### Effectiveness of Multicomponent Versus Standalone App Interventions

Although the aim of this review was to determine the effectiveness of mobile apps in promoting healthy behaviors, with 46% (6/13) of studies being multicomponent interventions, it is difficult to identify the unique contribution to behavior change associated with the app versus other intervention components. All 6 multicomponent intervention studies reported at least one significant outcome, whereas only 57% (4/7) of standalone intervention studies reported significant outcomes. This observation is consistent with the results of other systematic reviews of heathy behavior change interventions [10,47] and suggests that the inclusion of an app in a multicomponent intervention may result in greater effectiveness in achieving healthy behavior changes. In our review, multicomponent interventions tended to be longer (17 weeks to 6 months) than standalone interventions (1 hour to 12 weeks). Previous literature has noted the correlation between a longer follow-up period for multicomponent interventions and their efficacy [47]. Furthermore, given the difficulty in conducting intensive interventions (eg, on a daily basis) because of resource and time constraints, the inclusion of a mobile app in a multicomponent intervention may potentially serve as a tool that consistently motivates healthy behavior changes between intervention activities and study visits. However, results from multicomponent intervention studies should be interpreted with caution as they may be biased or underpowered [48].

The tendency for multicomponent interventions to be more efficient has been described previously [49]; however, few studies have directly investigated the effect of the individual components of a multicomponent intervention. Therefore, the effects of the multicomponent interventions reported in this review cannot be attributed solely to the inclusion of the apps. Other intervention components or combinations of components, such as intervention length, irrespective of app use, may have contributed to the reported intervention effects [50].

### App Features of Effective Interventions

The inclusion of certain features in an app may increase the effectiveness of the interventions [51]. The ability of an app to automatically collect and record health data using wirelessly connected devices such as accelerometers and scales may make it more convenient for users to keep track of their progress and receive continuous feedback and, thus, serve as an enabling factor for healthy behavior changes, as they facilitate personalized experiences based on users’ preferences and needs [52]. Furthermore, 30% (3/10) [24,25,32] of studies reporting at least one significant outcome incorporated the use of wireless technology to gather data from an external device directly to a mobile device. In accordance with the Fogg Behavior Model [53], decreasing barriers to use (via automatic data collection and data integration) may decrease the level of effort required by users and, in turn, increase the likelihood that users will engage in healthy behavior changes.

The incorporation of gamification in apps has previously been found to be associated with increased motivation and the establishment of long-lasting habits [54]. Approximately 71% (5/7) of studies that incorporated elements of gamification reported at least one significant healthy behavior outcome [28,29,31,33,36]. The analysis of health and fitness apps related to diet and physical activity by Lister et al [55] suggests that the use of gamification to increase motivation may only lead to temporary healthy behavior changes, as gamification often fails to address the individual’s *ability* and the *presence of triggers* (cues to prompt target behavior), which when combined with *motivation*, form the 3 elements of the Fogg Behavior Model [53]. Mobile app developers are encouraged to integrate key aspects of behavior change theories to promote healthy behavior changes; 75% (3/4) [28,29,31] of studies included in this review that incorporated gamification and at least one behavior change theory in its app design reported at least one significant healthy behavior outcome.

### Strengths and Limitations

This review was conducted under the guidance of a research librarian to ensure thoroughness of the search following PRISMA guidelines [16]. Study screening, data extraction, and risk of bias assessments were performed by at least two independent reviewers and discussed until consensus was reached. The narrow scope of this review provides a thorough overview of the literature to those interested in healthy behavior promotion studies targeting children that focused on mobile apps rather than other eHealth technologies such as SMS text messages and web-based technologies. Limitations are that because of the limited evidence base that is currently available, most studies included were quasi-experimental, and as evident from the risk of bias assessment, approximately half of the studies were rated as having a serious risk of bias. Even among the 4 RCTs, 3 (75%) were rated as having concerns regarding their risk of bias. Given that all but one of the studies included in this review were conducted in Western countries, this review may not be generalizable to the larger global community. It should also be noted that a meta-analysis was not completed because of the diverse nature of the outcomes and the reporting of the studies included in this review. Our results may be limited by our choice, the number of databases searched, and publication bias. Finally, we were unable to retrieve the relevant data specifically for the subgroups of children aged 8 to 12 years, as not all included studies reported the breakdown of participants’ ages, and thus, our assessment may be more generalizable to children outside this age group. Future studies should include a formal evaluation of behavior change theory application to measure the extent of theory application in mobile apps and intervention designs.

### Conclusions

The results of this systematic review suggest the potential of apps as components of healthy behavior promotion interventions to increase the adoption of healthy behaviors among children. However, the effectiveness of these mobile health interventions in improving anthropometric measures remains unclear. Dietary factors and physical activity measures emerged as the most common significant outcomes reported; gamification, wireless connection to external sensors, goal setting, and social support were common app features of interventions that reported significant outcomes. Further investigation is needed to determine the effectiveness of mobile apps as standalone interventions. With most of the literature comprising quasi-experimental studies that were relatively short in duration, future research in this area should use more rigorous study designs and be longer in duration (ie, >1 year) to truly generate a comprehensive understanding of the efficacy of mobile apps in healthy behavior promotion interventions for children.

## Appendix

Multimedia Appendix 1 Literature search strategy.

Multimedia Appendix 2 Data extraction template.

## Abbreviations

%BMI_p50_: percentage over BMI relative to the 50th percentile
%BMI_p95_: percentage over BMI relative to the 95th percentile
ATLAS: Active Teen Leaders Avoiding Screen-time
MVPA: moderate to vigorous physical activity
PRISMA: Preferred Reporting Items for Systematic Reviews and Meta-Analyses
RCT: randomized controlled trial
zBMI: BMI *z* score
