# Supplementary material for: Effectiveness of Mobile Apps in Promoting Healthy Behavior Changes and Preventing Obesity in Children: Systematic Review
Source: JMIR Pediatr Parent. 2022 Mar 28;5(1):e34967. doi: 10.2196/34967 (PMC9002598; doi:10.2196/34967)
Supplement: Multimedia Appendix 1 [file pediatrics_v5i1e34967_app1.docx]

## Multimedia Appendix 1

**Literature Search Strategy.**

| **Database** | **Search Terms** |
| --- | --- |
| **MEDLINE (Ovid)** | 1. (mhealth or ehealth).tw,kw. 2. (iterative develop* or co-creat*).tw,kw. 3. (mobile app* or software design or user-computer interface).tw,kw. 4. gamif*.tw,kw. 5. (interactivity or user experience or human-centered design).tw,kw. 6. (smartphone or tablet or iphone or android or ipad).tw,kw. 7. CHILD HEALTH/ or HEALTH BEHAVIOR/ or HEALTH PROMOTION/ or HEALTH/ or ADOLESCENT HEALTH/ 8. OBESITY MANAGEMENT/ or PEDIATRIC OBESITY/ 9. (health or obesity management or pediatric obesity).tw,kw. 10. Chronic Disease/ or chronic disease*.tw,kw. 11. (physical activity or physical fitness or exercise or active play).tw,kw. 12. healthy lifestyle/ or sedentary lifestyle/ 13. (sedentary or screen time).tw,kw. 14. Healthy Diet/ 15. healthy diet.tw,kw. 16. (vegetable* or fruit*).tw,kw. 17. (sugary drink* or sugary beverage*).tw,kw. 18. focus groups/ or "surveys and questionnaires"/ 19. preventive health services/ or "early intervention (education)"/ or health education/ 20. (focus group* or survey* or questionnaire* or preventive health or health education or health intervention).tw,kw. 21. or/1-6 22. or/7-17 23. or/18-20 24. and/21-23 25. limit 24 to yr="2008 - 2021" 26. exp child/ or exp "congenital, hereditary, and neonatal diseases and abnormalities"/ or exp infant/ or adolescent/ or exp pediatrics/ or child, abandoned/ or exp child, exceptional/ or child, orphaned/ or child, unwanted/ or minors/ or (pediatric* or paediatric* or child* or newborn* or congenital* or infan* or baby or babies or neonat* or pre-term or preterm* or premature birth* or NICU or preschool* or pre-school* or kindergarten* or kindergarden* or elementary school* or nursery school* or (day care* not adult*) or schoolchild* or toddler* or boy or boys or girl* or middle school* or pubescen* or juvenile* or teen* or youth* or high school* or adolesc* or pre-pubesc* or prepubesc*).mp. or (child* or adolesc* or pediat* or paediat*).jn. 27. 25 and 26 |
| **Embase (Ovid)** | 1. (mhealth or ehealth).tw,kw. 2. (iterative develop* or co-creat*).tw,kw. 3. mobile application/ or mobile app*.tw,kw. 4. software design.kw,tw. or software design/ 5. user-computer interface.tw,kw. or computer interface/ 6. gamif*.tw,kw. 7. (interactivity or user experience or human-centered design).tw,kw. 8. microcomputer/ or smartphone/ 9. (smartphone or tablet or iphone or android or ipad).tw,kw. 10. child health care/ or child health/ 11. health behavior/ 12. health education/ or health promotion/ or health program/ or prevention/ 13. obesity/ or adolescent obesity/ or childhood obesity/ 14. (health or obesity management or pediatric obesity).tw,kw. 15. chronic disease/ or chronic disease*.tw,kw. 16. exercise/ or physical activity/ or fitness/ 17. (physical activity or physical fitness or exercise or active play).tw,kw. 18. healthy lifestyle/ 19. sedentary lifestyle/ 20. (sedentary or screen time).tw,kw. 21. healthy diet/ or healthy diet.tw,kw. 22. (vegetable* or fruit*).tw,kw. 23. (sugary drink* or sugary beverage*).tw,kw. 24. focus group.tw,kw. 25. questionnaire*.tw,kw. or questionnaire/ 26. health survey/ or survey*.tw,kw. 27. preventive medicine/ or preventive health service/ 28. (preventive health or health education or health intervention).tw,kw. 29. or/1-9 30. or/10-21 31. or/24-28 32. and/29-31 33. limit 32 to yr="2008 - 2021" 34. exp child/ or exp "congenital, hereditary, and neonatal diseases and abnormalities"/ or exp infant/ or exp adolescence/ or exp infant, newborn/ or exp child, preschool/ or (pediatric* or paediatric* or child* or newborn* or congenital* or infan* or baby or babies or neonat* or pre-term or premature birth or NICU or preschool* or preschool* or kindergarten* or elementary school* or nursery school* or schoolchild* or toddler* or boy or boys or girl* or middle school* or pubescen* or juvenile* or teen* or youth* or high school* or adolesc* or pre-pubesc*).mp. or (child* or adolesc* or pediat* or paediat*).jn. 35. 33 and 34 |
| **PsycINFO (EBSCOhost)** | 1. AB(mhealth OR ehealth) OR TI(mhealth OR ehealth) 2. AB((iterative N3 develop*) OR co-creat* OR cocreat*) OR TI((iterative N3 develop*) OR co-creat* OR cocreat*) 3. (DE "Human Computer Interaction") 4. AB((mobile N3 app*) OR (software N3 design) OR (user-computer N3 interface)) OR TI((mobile N3 app*) OR (software N3 design) OR (user-computer N3 interface)) 5. AB(gamif*) OR TI(gamif*) 6. AB(interactivity OR (user N3 experience) OR (human-centered N3 design)) OR TI(interactivity OR (user N3 experience) OR (human-centered N3 design)) 7. AB(smartphone* OR tablet* OR iphone* OR android* OR ipad*) OR TI(smartphone* OR tablet* OR iphone* OR android* OR ipad*) 8. DE "Health Behavior" OR DE "Health Education" OR DE "Health Promotion" OR AB((child N3 health) OR (health N3 behavio#r) OR (health N3 promotion) OR (adolescent N3 health)) OR TI((child N3 health) OR (health N3 behavio#r) OR (health N3 promotion) OR (adolescent N3 health)) 9. DE "Sedentary Behavior" OR DE "Overweight" OR AB((obesity) or (sedentary)) OR TI((obesity) or (sedentary)) 10. DE "Chronic Illness" OR AB(chronic disease*) OR TI(chronic disease*) 11. DE "Physical Activity" OR DE "Active Living" OR DE "Physical Fitness" OR DE "Exercise" OR AB((physical N3 activity) or (physical N3 fitness) or (exercise) or (active N3 play)) OR TI((physical N3 activity) or (physical N3 fitness) or (exercise) or (active N3 play)) 12. DE "Screen Time" OR AB(screen N3 time) OR TI(screen N3 time) 13. AB(healthy diet*) OR TI(healthy diet*) 14. AB(vegetable* OR fruit*) OR TI(vegetable* OR fruit*) 15. AB(sugary drink* OR sugary beverage*) OR TI(sugary drink* OR sugary beverage*) 16. DE "Surveys" OR DE "Questionnaires" OR AB(survey* or questionnaire*) OR TI(survey* or questionnaire*) 17. AB(focus group*) OR TI(focus group*) 18. DE "Early Intervention" OR AB((preventive N3 health) or (health N3 intervention)) OR TI((preventive N3 health) or (health N3 intervention)) 19. DE "Health Education" OR AB(health N3 education) OR TI(health N3 education) 20. S1 OR S2 OR S3 OR S4 OR S5 OR S6 OR S7 21. S8 OR S9 OR S10 OR S11 OR S12 OR S13 OR S14 OR S15 22. S16 OR S17 OR S18 OR S19 23. S20 AND S21 AND S22   Limiters - Published Date: 20080101-20210703; Age Groups: Childhood (birth-12 yrs), Neonatal (birth-1 mo), Infancy (2-23 mo), Preschool Age (2-5 yrs), School Age (6-12 yrs), Adolescence (13-17 yrs)  Search modes = Boolena/Phrase |
| **CINAHL Complete (EBSCOhost)** | 1. (MH "Mobile Applications") OR AB(mobile N3 app*) OR TI(mobile N3 app*) 2. AB(mhealth OR ehealth) OR TI(mhealth OR ehealth) 3. AB((iterative N3 develop*) OR co-creat* OR cocreat*) OR TI((iterative N3 develop*) OR co-creat* OR cocreat*) 4. (MH "User-Computer Interface+") OR (MH "Smartphone") OR AB((user-computer N3 interface) or smartphone) OR TI((user-computer N3 interface) or smartphone) 5. (MH "Software Design") or AB(software N3 design) OR TI(software N3 design) 6. AB(gamif*) OR TI(gamif*) 7. AB(interactivity OR (user N3 experience) OR (human-centered N3 design)) OR TI(interactivity OR (user N3 experience) OR (human-centered N3 design)) 8. (MH "Computers, Portable+") OR AB(tablet*) OR TI(tablet*) 9. AB(iphone* OR android* OR ipad*) OR TI(iphone* OR android* OR ipad*) 10. (MH "Health Behavior+") or AB((health N3 behavio#r) or (child N3 health) or (health or adolescent N3 health)) OR TI((health N3 behavio#r) or (child N3 health) or (health or adolescent N3 health)) 11. (MH "Health Promotion+") OR AB(health N3 promotion) OR TI(health N3 promotion) 12. (MH "Pediatric Obesity") OR (MH "Attitude to Obesity") OR AB(obesity N3 management) OR TI(obesity N3 management) 13. (MH "Life Style, Sedentary") OR AB(sedentary) OR TI(sedentary) 14. (MH "Chronic Disease") OR AB(chronic disease*) OR TI(chronic disease*) 15. (MH "Physical Activity") OR AB((physical N3 activity) or (active N3 play)) OR TI((physical N3 activity) or (active N3 play)) 16. (MH "Physical Fitness+") OR AB(physical N3 fitness) OR TI(physical N3 fitness) 17. (MH "Exercise+") OR AB(exercise*) OR TI(exercise*) 18. AB(screen N3 time) OR TI(screen N3 time) 19. AB(healthy N3 diet) OR TI(healthy N3 diet) 20. (MH "Vegetables+") OR AB(vegetable*) OR TI(vegetable*) 21. (MH "Fruit+") OR AB(fruit*) OR TI(fruit*) 22. (MH "Carbonated Beverages") OR AB((sugary N3 drink*) or (sugary N3 beverage*)) OR TI((sugary N3 drink*) or (sugary N3 beverage*)) 23. (MH "Surveys+") OR (MH "Survey Research") OR AB(survey*) OR TI(survey*) 24. (MH "Structured Questionnaires") OR (MH "Open-Ended Questionnaires") OR (MH "Questionnaires+") OR AB(questionnaire*) OR TI(questionnaire*) 25. (MH "Focus Groups") OR AB(focus N3 group*) or TI(focus N3 group*) 26. (MH "Health Education+") OR (MH "Student Health Education") OR AB(health N3 education) OR TI(health N3 education) 27. (MH "Early Intervention+") OR (MH "Intervention Trials") OR AB((preventive N3 health) OR (health N3 education) or (health N3 intervention)) OR TI((preventive N3 health) or (health N3 education) or (health N3 intervention)) 28. (pediatric* or paediatric* or child* or newborn* or congenital* or infan* or baby or babies or neonat* or “pre-term” or preterm or “premature birth*” or NICU or preschool* or “preschool*” or kindergarten* or “elementary school*” or “nursery school*” or schoolchild* or toddler* or boy or boys or girl* or “middle school*” or pubescen* or juvenile* or teen* or youth* or “high school*” or adolesc*or prepubesc* or “pre-pubesc*” or "(MH "Child+") OR (MH "Adolescence+") OR (MH "Minors (Legal)") or "(MH "Child Abuse, Sexual") OR (MH "Child Behavior Disorders+") OR (MH "Child, Medically Fragile") OR (MH "Child Day Care") OR (MH "Child Behavior+") OR (MH "Child Mortality") OR (MH "Child Passenger Safety") OR (MH "Child Development Disorders, Pervasive+") OR (MH "Child Custody") OR (MH "Child Abuse+") OR (MH "Child Nutritional Physiology+") OR (MH "Child Behavior Checklist") ) OR SO ( child* or pediatric* or paediatric* or adolescent ) 29. S1 OR S2 OR S3 OR S4 OR S5 OR S6 OR S7 OR S8 OR S9 30. S10 OR S11 OR S12 OR S13 OR S14 OR S15 OR S16 OR S17 OR S18 OR S19 OR S20 OR S21 OR S22 31. S23 OR S24 OR S25 OR S26 OR S27 32. S29 AND S30 AND S31 33. S28 AND S32   Limiters - Published Date: 20080101-20210703  Search modes = Boolena/Phrase |
| **ERIC (EBSCOhost)** | 1. (MH "Mobile Applications") OR AB(mobile N3 app*) OR TI(mobile N3 app*) 2. AB(mhealth OR ehealth) OR TI(mhealth OR ehealth) 3. AB((iterative N3 develop*) OR co-creat* OR cocreat*) OR TI((iterative N3 develop*) OR co-creat* OR cocreat*) 4. (MH "User-Computer Interface+") OR (MH "Smartphone") OR AB((user-computer N3 interface) or smartphone) OR TI((user-computer N3 interface) or smartphone) 5. (MH "Software Design") or AB(software N3 design) OR TI(software N3 design) 6. AB(gamif*) OR TI(gamif*) 7. AB(interactivity OR (user N3 experience) OR (human-centered N3 design)) OR TI(interactivity OR (user N3 experience) OR (human-centered N3 design)) 8. (MH "Computers, Portable+") OR AB(tablet*) OR TI(tablet*) 9. AB(iphone* OR android* OR ipad*) OR TI(iphone* OR android* OR ipad*) 10. (MH "Health Behavior+") or AB((health N3 behavio#r) or (child N3 health) or (health or adolescent N3 health)) OR TI((health N3 behavio#r) or (child N3 health) or (health or adolescent N3 health)) 11. (MH "Health Promotion+") OR AB(health N3 promotion) OR TI(health N3 promotion) 12. (MH "Pediatric Obesity") OR (MH "Attitude to Obesity") OR AB(obesity N3 management) OR TI(obesity N3 management) 13. (MH "Life Style, Sedentary") OR AB(sedentary) OR TI(sedentary) 14. (MH "Chronic Disease") OR AB(chronic disease*) OR TI(chronic disease*) 15. (MH "Physical Activity") OR AB((physical N3 activity) or (active N3 play)) OR TI((physical N3 activity) or (active N3 play)) 16. (MH "Physical Fitness+") OR AB(physical N3 fitness) OR TI(physical N3 fitness) 17. (MH "Exercise+") OR AB(exercise*) OR TI(exercise*) 18. AB(screen N3 time) OR TI(screen N3 time) 19. AB(healthy N3 diet) OR TI(healthy N3 diet) 20. (MH "Vegetables+") OR AB(vegetable*) OR TI(vegetable*) 21. (MH "Fruit+") OR AB(fruit*) OR TI(fruit*) 22. (MH "Carbonated Beverages") OR AB((sugary N3 drink*) or (sugary N3 beverage*)) OR TI((sugary N3 drink*) or (sugary N3 beverage*)) 23. (MH "Surveys+") OR (MH "Survey Research") OR AB(survey*) OR TI(survey*) 24. (MH "Structured Questionnaires") OR (MH "Open-Ended Questionnaires") OR (MH "Questionnaires+") OR AB(questionnaire*) OR TI(questionnaire*) 25. (MH "Focus Groups") OR AB(focus N3 group*) or TI(focus N3 group*) 26. (MH "Health Education+") OR (MH "Student Health Education") OR AB(health N3 education) OR TI(health N3 education) 27. (MH "Early Intervention+") OR (MH "Intervention Trials") OR AB((preventive N3 health) OR (health N3 education) or (health N3 intervention)) OR TI((preventive N3 health) or (health N3 education) or (health N3 intervention)) 28. S1 OR S2 OR S3 OR S4 OR S5 OR S6 OR S7 OR S8 OR S9 29. S10 OR S11 OR S12 OR S13 OR S14 OR S15 OR S16 OR S17 OR S18 OR S19 OR S20 OR S21 OR S22 30. S23 OR S24 OR S25 OR S26 OR S27 31. S28 AND S29 AND S30   Limiters - Published Date: 20080101-20210703; Education Level: Early Childhood Education, Elementary Education, Grade 1, Grade 2, Grade 3, Grade 4, Grade 5, Grade 6, Grade 7, Kindergarten, Primary Education   Search modes - Boolean/Phrase |
| **Grey Literature** | **Clinicaltrials.gov:** obesity, childhood; mobile health  **ProQuest Dissertations and Theses Global:** (ab(childhood obesity) OR ti (childhood obesity)) AND (ti(mobile) OR ab(mobile))  **Google Scholar**: (childhood obesity) AND (mobile health), first 100 results |
