# Supplementary material for: Effectiveness of Mobile Apps in Promoting Healthy Behavior Changes and Preventing Obesity in Children: Systematic Review
Source: JMIR Pediatr Parent. 2022 Mar 28;5(1):e34967. doi: 10.2196/34967 (PMC9002598; doi:10.2196/34967)
Supplement: Multimedia Appendix 2 [file pediatrics_v5i1e34967_app2.docx]

## Multimedia Appendix 2

**Data extraction template.**

| **Study Details** | |
| --- | --- |
|  | - Title - Author(s) - Year |
| **Study Characteristics** | |
|  | - Study design type - Study duration - Mobile app features - Intervention design - Multicomponent/standalone intervention - Behaviour change theory |
| **Study Sample** | |
|  | - Inclusion/exclusion criteria - Sample size - Sociodemographic characteristics   - Age   - Sex   - Ethnicity/race   - Socioeconomic status   - Risk of obesity |
| **Results** | |
|  | - Outcome measures   - Anthropometry: BMI, BMI z-score, BMI percentile, waist circumference, body fat percentage   - Physical activity: moderate to vigorous physical activity, step count, metabolic equivalents, physical strength, fitness, attitudes and perceptions of physical activity   - Dietary: fruits and vegetables consumption, sugar-sweetened beverage consumption, breakfast likeliness, attitudes and perceptions of diet   - Screen time   - Process evaluation - App evaluation (if applicable) |
